# Sequential high-content profiling of the IgG-autoantibody repertoire reveals novel antigens in rheumatoid arthritis

**DOI:** 10.1186/s13075-016-1135-6

**Published:** 2016-10-12

**Authors:** Stefan Vordenbäumen, Angelika Lueking, Petra Budde, Hans-Dieter Zucht, Heike Goehler, Ralph Brinks, Rebecca Fischer-Betz, Jutta Richter, Ellen Bleck, Jacqueline Detert, Hans-Eckhard Langer, Anne Sörgel, Gerd-Rüdiger Burmester, Peter Schulz-Knappe, Matthias Schneider

**Affiliations:** 1 Department Rheumatology & Hiller Research Unit Rheumatology, Heinrich-Heine-University, Moorenstr. 5, Düsseldorf, 40225 Germany; 2 Protagen AG, Dortmund, Germany; 3 German Diabetes Center, Institute for Biometry and Epidemiology, Düsseldorf, Germany; 4 Department Rheumatology and Clinical Immunology, Charité – University Medicine Berlin, Berlin, Germany; 5 Rheumatology, Clinical Immunology and Osteololgy at Evangelisches Krankenhaus, Düsseldorf, Germany

**Keywords:** Rheumatoid arthritis, Autoantibody, Biomarker

## Abstract

**Background:**

The aim was to identify novel diagnostic autoantibody candidates for rheumatoid arthritis (RA) by comprehensive screening for autoreactivity.

**Method:**

We incubated 5892 recombinant proteins coupled to fluorescent beads, with patients’ sera for the detection of IgG-autoantibodies in three independent patient cohorts: A (n = 72 patients with established RA); B/B- (n = 116 patients with early RA (B) and n = 51 CCP-negative patients with early RA from B (B-)); and C (n = 184 patients with early seronegative RA), in comparison to matched healthy controls. Intersects of significantly increased autoantibodies as determined by the Mann-Whitney test were sought.

**Result:**

Screening of 5892 antigens in RA cohorts A and B, or the seronegative cohorts B- and C revealed intersects of 23 and 13 significantly increased autoantibodies, respectively. Reactivity to three antigens was increased in all cohorts tested: N-acetylglucosamine-1-phosphate transferase, gamma subunit (GNPTG), heterogeneous nuclear ribonucleoprotein A1-like 2 (HNRNPA1), and insulin-like growth factor binding protein 2 (IGFBP2).

**Conclusions:**

Comprehensive sequential screening for autoantibodies reveals novel candidates for diagnostic markers in both seropositive and seronegative RA and suggests new fields of research into the pathogenesis of RA.

## Background

Rheumatoid arthritis (RA) is a chronic inflammatory autoimmune disease characterized by destruction of cartilage and bone. RA pathogenesis features numerous autoimmune processes such as autoreactive T cells and the formation of autoantibodies [1]. Amongst the autoantibodies present in RA sera, rheumatoid factor (RF) and antibodies against citrullinated peptides (ACPA) have emerged as important diagnostic and prognostic markers [2], although a range of other autoantibodies have been identified [3]. Evidence is accumulating that RF and ACPA are directly involved in the pathogenesis of the disease [4], potentially even prior to the occurrence of synovial inflammation [5]. Despite progress in the serologic diagnosis of RA introduced by the detection of these antibodies, a considerable number of patients with RA do not display RF and/or ACPA [6, 7]. In these cases, the diagnosis of RA relies heavily upon clinical presentation and imaging procedures. Thus, identification of reliable additional markers to recognize RF/ACPA-negative RA and to further ascertain the diagnosis of RF/ACPA-positive RA is desirable.

We adopted a multiplex bead-based approach to sequentially screen sera from three independent patient cohorts: cohort A, patients with established RA; cohort B, patients with early RA; and cohort C, patients with seronegative RA) for the presence of autoantibodies to over 5800 human antigens, aiming to (1) retrieve a collection of candidates for future diagnostic assay development, and (2) gain new insights into the pathogenesis of RA.

## Methods

### Sample cohorts and autoantibody identification strategy

In order to identify potential novel diagnostic autoantibodies, antibody profiles of serum samples from different RA patient cohorts were sequentially compared to healthy controls. Healthy controls were chosen in an age-adjusted and sex-adjusted manner from specimens from blood donors of the Bavarian Red Cross, Germany or collected from healthy individuals after the exclusion of rheumatic diseases from a community screening program. Patient cohorts were as follows. Cohort A (established RA) comprised 72 consecutive patients with established RA according to American College of Rheumatology (ACR)/European League Against Rheumatism (EULAR) 2010 criteria (age 56.1 ± 13.3 years, 73.6 % female, Disease Activity Score for 28 joints (DAS28) 3.5 ± 2.3, therapy: methotrexate 40 %, leflunomide 12.5 %, tumor necrosis factor alpha (TNFa)-blockade 18 %) from the outpatient department of Heinrich-Heine-University Düsseldorf. These patients were compared to 71 age-matched and sex-matched healthy controls (age 54.6 ± 11.3 years, 73.2 % female). Cohort B (early RA) comprised 116 patients with early RA from the HIT HARD study [8] (age 49.8 ± 13.8 years, 71.3 % female, DAS28 6.1 ± 1.0, all therapy-naive). These patients were compared to 116 healthy controls (age 49.8 ± 12.8 years, 71.6 % female). A subgroup analysis was conducted in seronegative patients from cohort B, termed B- (n = 51, age 54.5 ± 13.3 years, 66 % female). Cohort C (seronegative cohort) comprised 184 patients with ACPA-negative RA according to 2010 ACR/EULAR criteria (age 60.2 ± 13.8 years, 62.5 % % female, all therapy- naive). These patients were compared to 184 healthy controls (age 55.2 ± 10 years, 62.5 % female). All serum samples were obtained by standard procedures and stored at -80 °C until use.

### Multiplex bead-based autoantibody detection

We produced 5892 recombinant antigens in *Escherichia coli* and purified them. Five cDNA libraries originating from different human tissues (fetal brain, colon, lung, liver, Cd4 induced and non-induced T cells) were used for the recombinant production of human antigens. All of these cDNA libraries were oligo(dT)-primed, containing the coding region for an N-terminally located hexa-histidine-tag and were under transcriptional control of the lactose inducible promoter from *E. coli* [9]. Sequence integrity of the cDNA libraries was confirmed by 5’ DNA sequencing. Additionally, expression clones representing the full-length sequence derived from the human ORFeome collection [10] were included. Individual antigens were designed *in silico*, synthesized chemically (Life Technologies, Carlsbad, USA) and cloned into the expression vector pQE30-NST fused to the coding region for the N-terminal-located His6-tag. Of the antigens, 73 % were produced by cDNA library expression clones, 24 % of the antigens derive from clones of the human ORFeome collection and 3 % of the antigens were based on *in silico* design.

Recombinant gene expression was performed in *E. coli* SCS1 cells carrying plasmid pSE111 for improved expression of human genes [11]. Cells were cultivated in 200 ml auto-induction medium (Overnight Express auto-induction medium, Merck, Darmstadt, Germany) overnight and harvested by centrifugation. Bacterial pellets were lysed by resuspension in 15 ml lysis buffer (6 M guanidinium-HCl, 0.1 M NaH_2_PO_4_, 0.01 M Tris-HCl, pH 8.0). Soluble proteins were affinity-purified after binding to Protino® Ni-IDA 1000 Funnel Column (Macherey-Nagel, Düren, Germany). Columns were washed with 8 ml washing buffer (8 M urea, 0.1 M NaH_2_PO_4_, 0.01 M Tris-HCl, pH 6.3). Proteins were eluted in 3 ml elution buffer (6 M urea, 0.1 M NaH_2_PO_4_, 0.01 M Tris-HCl, 0.5 % (w/v) trehalose pH 4.5). Each protein preparation was transferred into 2D-barcoded tubes, lyophilized and stored at -20 °C.

In this study, twenty different bead-based arrays containing up to 384 different proteins were used. For production of bead-based arrays (BBA), the proteins were coupled to magnetic carboxylated color-coded beads (MagPlex™ microspheres, Luminex Corporation, Austin, TX, USA). The manufacturer’s protocol for coupling proteins to MagPlex™ microspheres was adapted to use liquid handling systems. A semi-automated coupling procedure of one BBA encompassed 384 single, separate coupling reactions, which were carried out in four 96-well plates. For each single coupling reaction, up to 12.5 μg antigen and 8.8 × 10^5^ MagPlex™ beads of one color region (ID) were used. All liquid handling steps were carried out by either an eight-channel pipetting system (Starlet, Hamilton Robotics, Bonaduz, Switzerland) or a 96-channel pipetting system (Evo Freedom 150, Tecan, Männderdorf, Switzerland). For semi-automated coupling, antigens were dissolved in H_2_O, and aliquots of 60 microliters were transferred from 2D barcode tubes to 96-well plates. MagPlex™ microspheres were homogeneously resuspended and each bead ID was transferred in one well of a 96-well plate. The 96-well plates containing the microspheres were placed on a magnetic separator (LifeSep™, Dexter Magnetic Technologies Inc., Elk Grove Village, USA) to sediment the beads for washing steps and on a microtiter plate shaker (MTS2/4, IKA) to facilitate permanent mixing for incubation steps.

For coupling, the microspheres were washed three times with activation buffer (100 mM NaH_2_PO_4_, pH 6.2) and resuspended in 120 μl activation buffer. To obtain reactive sulfo-NHS-ester intermediates, 15 μl 1-ethly-3-(3-dimethlyaminopropyl) carbodiimide (50 mg/ml) and 15 μl N-hydroxy-succinimide (50 mg/ml) were applied to microspheres. After 20 minutes incubation (900 rpm, room temperature (RT)) the microspheres were washed three times with coupling buffer (50 mM MES, pH 5.0) and resuspended in 65 μl coupling buffer. Immediately, 60 μl antigen solution was added to reactive microspheres and coupling took place over 120 minutes under permanent mixing (900 rpm, RT). After three wash cycles using washing buffer (PBS, 0.1 % Tween20) coupled beads were resuspended in blocking buffer (PBS, 1 % BSA, 0.05 % ProClin300), incubated for 20 minutes (900 rpm, RT) and then transferred to be maintained at 4–8 °C for 12–72 h.

To monitor the assay performance various proteins were used as control proteins and coupled individually to microspheres as described for antigens. Human and mouse IgG (Sigma-Aldrich, St. Louis, USA) were used to control the reactivity of the detection antibodies, BSA (Sigma-Aldrich) to monitor the background and *E. coli* lysate to detect serum antibodies directed against *E. coli* proteins. For coupling reaction control 10 μg, 25 μg, 100 μg and 200 μg human IgG, 200 μg mouse IgG, 1 μg BSA and 500 μg *E. coli* proteins were used, respectively. A bead mix of a multiplex BBA was generated by pooling 384 antigen-coupled beads together with control protein-coupled beads. The BBA was stored at 4–8 °C until use.

Serum samples were transferred to 2D barcode tubes and a 1:100 serum dilution was prepared with assay buffer (PBS, 0.5 % BSA, 10 % *E. coli* lysate, 50 % Low-Cross buffer (Candor Technologies, Nürnberg, Germany)) in 96-well plates. The serum dilutions were first incubated for 20 minutes to neutralize any human IgG eventually directed against *E. coli* proteins. The BBA was sonicated for 5 minutes and the bead mix was distributed in 96-well plates. After three wash cycles with washing buffer (PBS, 0.05 % Tween20) serum dilutions (50 μl) were added to the bead mix and incubated for 20 h (900 rpm, 4–8 °C). Supernatants were removed from the beads by three wash cycles, and secondary R-phycoerythrin-labeled antibody (5 μg/ml, goat anti-human, Dianova, Hamburg, Germany) was added for a final incubation of 45 minutes (900 rpm, RT). The beads were washed three times with washing buffer (PBS, 0.1 % Tween20) and resuspended in 100 μl sheath fluid (Luminex Corporation). Subsequently, beads were analyzed in a FlexMap3D device for fluorescent signal readout (DD gate 7.500–15.000; sample size: 80 μl; 1000 events per bead ID; timeout 60 sec). The binding events were displayed as median fluorescence intensity (MFI). Measurements were disregarded when low numbers of bead events (<30 beads) were counted per bead ID.

### Statistical analysis

Our antigen prioritization approach was sequential performance of the Mann-Whitney test to identify intersects of significantly upregulated markers in the patient cohorts compared to matched healthy controls. For data pre-processing and normalization, if fewer than 30 beads were counted for a specific antigen, the corresponding MFI value was set to missing. Samples or antigens were discarded from further analysis if either a patient sample or an antigen had >20 % missing values. In total, six patients and 1.5 % of antigens tested were discarded because of >20 % missing values. Prior to normalization, MFI values were log2-transformed. Quantile normalization was used to normalize data on each individual plate by BBA according to [12]. A missing MFI value for an antigen was replaced by the respective median value across all samples for this specific antigen. Replacement of the data by median imputed values was necessary for <0.25 % without notable accumulation in certain antigens. The target variable for all statistical work was the MFI of the detection antibody.

Antigens with an upregulated MFI and fold change ≥1 were further considered. The fold change was calculated as the ratio of median MFI of cases vs. controls. In each cohort, receiver operating characteristic (ROC) analyses were subsequently applied to calculate sensitivity at predefined 90 % specificity in comparison to respective matched healthy controls. The statistical software R (version 2.14.2 (2012-02-29)) [http://www.r-project.org] was used for all analyses.

## Results

### Candidate autoantibody identification in patients with RA

**Fig. 1:**
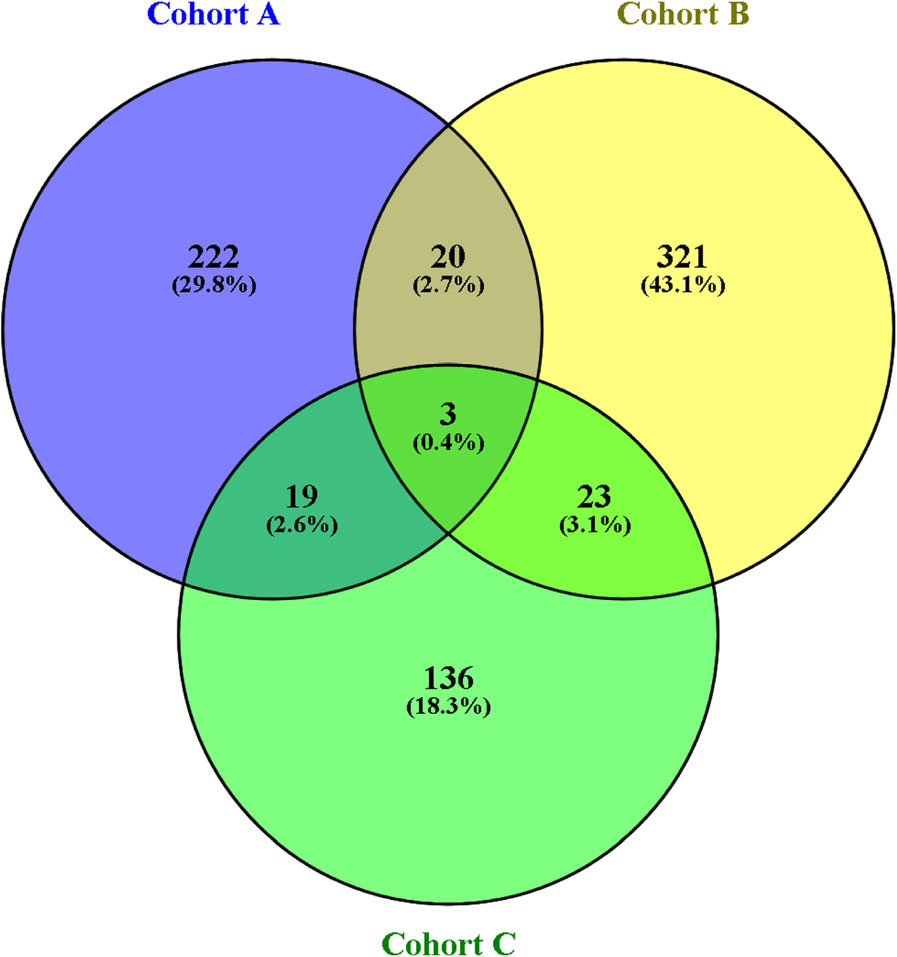
Intersects of significantly increased IgG-autoantibodies

Comparison of autoreactivity to 5892 antigens in sera from 72 patients with established RA (cohort A) and 116 therapy-naive patients with early RA (cohort B) resulted in an intersect of 23 antigens with an individual *p* value <0.05 and fold change >1 (Fig. 1). Fold changes ranged from 1.1 to 2.7 as detailed in Table 1.

**Table 1:** Intersect of significantly increased IgG-autoantibodies in patients with established RA (cohort A) and early RA (cohort B)

Antigen			Cohort A			Cohort B		
symbol	Name	Gene ID	*P*	FC	S	*P*	FC	S
DCTN1	Dynactin 1	1639	0.026	1.5	14	0.001	1.5	19
GNPTG	N-acetylglucosamine-1-phosphate transferase	84572	0.021	1.2	17	0.001	1.3	13
HNRNPA1	Heterogeneous nuclear ribonucleoprotein A1-like 2	144983	0.020	1.3	22	0.000	1.3	34
ITFG3	Integrin alpha FG-GAP repeat containing 3	83986	0.048	1.1	19	0.014	1.1	23
APOA4	Apolipoprotein A-IV	337	0.025	1.1	35	0.001	1.2	11
CCDC136	Coiled-coil domain containing 136	64753	0.039	2.2	14	0.031	1.7	2
CKAP4	Cytoskeleton-associated protein 4	10970	0.027	1.2	22	0.000	1.3	28
CLCN2	Chloride channel 2	1181	0.037	1.3	19	0.016	1.2	10
DMTF1	Cyclin D binding myb-like transcription factor 1	9988	0.047	1.3	22	0.022	1.3	11
FAM59B	Family with sequence similarity 59. member B	150946	0.047	1.9	18	0.000	1.5	15
IGFBP2	Insulin-like growth factor binding protein	3485	0.015	1.2	19	0.000	1.5	13
TMCO7	Transmembrane and coiled-coil domains 7	79613	0.009	1.1	21	0.000	1.5	20
USP48	Ubiquitin specific peptidase 48	84196	0.001	2.7	18	0.034	1.7	11
VIM	Vimentin	7431	0.022	1.3	19	0.000	1.7	38
ZFAND2B	Zinc finger. AN1-type domain 2B	130617	0.034	1.2	18	0.002	1.3	22
ATP6V1A	ATPase. H+ transporting. lysosomal 70 kDa. V1 subunit A	523	0.048	1.2	21	0.042	1.3	11
GSN	Gelsolin	2934	0.008	1.4	26	0.001	1.4	12
GSPT2	G1 to S phase transition 2	23708	0.045	1.2	14	0.043	1.3	8
HSBP1	Heat shock factor binding protein 1	3281	0.021	1.2	25	0.002	1.3	19
NONO	Non-POU domain containing. octamer-binding	4841	0.010	1.4	21	0.016	1.3	17
PRAP1	Proline-rich acidic protein 1	118471	0.030	1.3	14	0.035	1.6	9
YES1	YES proto-oncogene 1. Src family tyrosine kinase	6714	0.032	1.1	28	0.010	1.2	13
SSB	Sjogren syndrome antigen B (autoantigen La)	6741	0.009	1.4	15	0.042	1.1	17

Cohort A: established rheumatoid arthritis (RA) (n = 72). Cohort B: early RA (n = 116). *P* is *p* value for fold change (FC) and sensitivity (S) at 90 % specificity compared to matched healthy controls, according to the Mann-Whitney test

### Candidate autoantibody identification in seronegative patients with RA

In order to more specifically assess autoantibodies in seronegative RA, intersects of significantly increased autoantibodies in the seronegative subgroup of cohort B (termed B-) and an independent therapy-naïve cohort of 184 seronegative patients with RA (cohort C) was sought, resulting in 13 antigenic targets. Fold changes ranged from 1.1 to 1.8 as outlined in Table 2.

**Table 2:** Intersect of significantly increased IgG-autoantibodies in independent cohorts of patients with seronegative RA (cohorts B- and C)

Antigen			Cohort B-			Cohort C		
Symbol	Name	Gene ID	*P*	FC	S	*P*	FC	S
GNPTG	N-acetylglucosamine-1-phosphate transferase. gamma subunit	84572	0.001	1.4	16	0.04	1.1	10
HNRNPA1	Heterogeneous nuclear ribonucleoprotein A1-like 2	144983	0.000	1.4	41	0.000	1.1	24
ACTB	Actin beta	60	0.004	1.1	20	0.000	1.2	27
HSPD1	Heat shock 60 kDa protein 1 (chaperonin)	3329	0.008	1.6	14	0.002	1.8	17
PER1	Period homolog 1	5187	0.000	2.2	24	0.001	1.5	13
SMARCD3	SWI/SNF related. matrix associated. actin dependent regulator of Chromatin. subfamily d. member 3	6604	0.016	1.3	14	0.016	1.1	14
SMYD2	SET and MYND domain containing 2	56950	0.007	1.6	10	0.05	1.4	5
IGFBP2	Insulin-like growth factor binding protein 2. 36 kDa	3485	0.003	1.5	16	0.03	1.1	14
KLKB1	Kallikrein B. plasma (Fletcher factor) 1	3818	0.03	1.2	10	0.03	1.2	13
GON4L	Gon-4-like (C. elegans)	54856	0.01	1.2	12	0.02	1.1	15
CHMP5	Chromatin modifying protein 5	51510	0.03	1.5	12	0.004	1.3	14
DHX15	DEAH (Asp-Glu-Ala-His) box polypeptide 15	1665	0.007	1.5	12	0.03	1.1	11
FAHD2A	Fumarylacetoacetate hydrolase domain containing 2A	51011	0.04	1.2	16	0.05	1.2	14

Cohort B-: seronegative rheumatoid arthritis (RA) (n = 51). Cohort C: patients with seronegative RA (n = 184). *P* is *p* value for fold change (FC) and sensitivity (S) at 90 % specificity compared to matched healthy controls, according to the Mann-Whitney test

### Candidate autoantibody identification in all cohorts

Significantly increased autoantibodies in all cohorts were then identified resulting in diverse intersects as shown in Fig. 1. Significantly increased autoantibodies to three antigens were noted in all three cohorts (A, B and C) with fold changes ranging from 1.1 to 1.5 as detailed in Table 3.

**Table 3:** Intersect of significantly increased IgG autoantibodies in patients with RA (cohorts A, B, and C)

Antigen			Cohort A			Cohort B			Cohort C		
symbol	Name	Gene ID	*P*	FC	S	*P*	FC	S	*P*	FC	S
GNPTG	N-acetylglucosamine-1-phosphate transferase	84572	0.021	1.2	17	0.001	1.3	13	0.04	1.1	10
HNRNPA1	Heterogeneous nuclear ribonucleoprotein A1-like 2	144983	0.020	1.3	22	0.000	1.3	34	0.001	1.1	24
IGFBP2	Insulin-like growth factor binding protein 2	3485	0.01	1.2	19	0.000	1.5	13	0.03	1.1	15

Cohort A: patients with established rheumatoid arthritis (RA) (n = 72). cohort B: early RA (n = 116). cohort C: early seronegative RA (n = 184). *P* is *p* value for fold change (FC) and sensitivity (S) at 90 % specificity compared to matched healthy controls, according to the Mann-Whitney test

## Discussion

In the current study, we set out to identify new diagnostic markers in RA with the use of an extensive screening for IgG autoantibodies. The strength of this study consists in the comprehensive inclusion of a vast number of potential autoantigens and the subsequent narrowing of the candidate list, employing independent patient cohorts. Importantly, cohorts B and C represented sera from therapy-naïve patients with early RA, emphasizing the potential significance of our results for early serological diagnosis of the disease. Moreover, parts of the analyses focused on seronegative patients, resulting in identification of new interesting targets.

The risk of over-fitting the data is inherent in the adopted discovery approach with consideration of over 5800 antigens. Larger patient numbers were restricted due to practicability and cost considerations. Thus, we concentrated on single antigen performance and had to refrain from testing marker combinations. Furthermore, correction for multiple testing would have rendered the discovery approach unpromising a priori. Rather than direct diagnostic implications, the reported antigens are therefore considered attractive candidates, subject to improved ELISA-based assay development and subsequent clinical testing. The advantages of improved laboratory diagnosis are evident, because 30 % of the RA patient population cannot be identified applying diagnostic testing for ACPA and/or rheumatoid factor (RF) [8].

We and others assume that a certain percentage of seronegative patients are truly seronegative, i.e. do not generate autoantibodies in the course of the disease, whereas other patients have autoantibodies against so far undisclosed antigens [13]. We further expect that this group of false-negative seronegative patients is heterogeneous, and that detection of low-frequency autoantibodies is required for serological detection. Only unmodified antigens were tested and only IgG antibodies were considered in the current study. Modifications such as citrullination and carbamylation [14] carry the potential for better detection rates and may yet unmask a greater proportion of seronegative patients with RA. However, we chose to use non-modified proteins (i.e. without citrullination, carbamylation or acetylation) as we follow the hypothesis that RA may have several different pathophysiological routes, of which the formation of autoantibodies against post-translational modifications and protein complexes is just one (major) route. We find support for this hypothesis in the previous discovery of non-modified antigens such as RA33 (HNRNPA2/B1) and 14-3-3 [15, 16]. Interestingly, 14-3-3 is an antigen both in unmodified and in citrullinated form [17].

Similarly, we found vimentin in its native form (as confirmed by tandem mass spectrometry) to be an autoantigen in both cohorts A and B [18], and vimentin is known to be a major target of ACPA in its citrullinated form [19]. We further assumed that novel autoantibodies would most likely be present in smaller subsets of patients. Low prevalence is a common feature of autoantibodies in other autoimmune diseases: in systemic sclerosis several antigens have been described, which occur with a prevalence between 5 and 10 % (Th/T0, U3RNP, PM/Scl) [20], and in systemic lupus over 100 autoantibodies have been published, many of which have a prevalence below 15 % [21]. This makes their discovery difficult, because the ratio between high numbers of antigens used in omics-type screening is already in imbalance to the number of available patient samples. This imbalance is further complicated if novel markers are present in only 5–15 % of cases. Of note, in the present study, sensitivity of antigens across all group comparisons at a predefined specificity of 90 % was 17.7 ± 7.5 %, ranging from 2 to 41 %. Even though the study was designed to identify targets rather than determine their individual diagnostic performance, these results seem promising for individual diagnostic assay development. Furthermore, future validation of these diagnostic candidates should be carried out, ideally in early RA and include disease controls such as psoriatic arthritis.

Even though we used both established RA (cohorts A) and early RA (cohort B and C) for marker identification with the aim of identifying ubiquitous antigens in RA, the use of more homogeneous cohorts (e.g. seronegative patients only, or patients with early RA only) would represent an alternative approach to antigenic target identification. Clearly, the population used for screening approaches largely influences the resulting candidates. Thus, we maintain that an antibody identification strategy by an omics-type approach should be regarded as a means to reduce the number of potential individual targets. This in turn enables targeted individual autoantibody validation studies. We found support for this approach in other screening studies which similarly resulted in, albeit different, sets of potential targets [13]. This is probably due to different patient populations in terms of numbers of patients and ethnicity, and differences in the autoantibody identification approach [13], amongst other reasons. Nevertheless, all screening approaches result in potential targets for further individual testing and carry the potential for future diagnostic improvements, as has elegantly been demonstrated previously [13, 22].

After comprehensive screening of over 5800 antigens, we describe 23 potential new diagnostic antigens in RA and 13 for seronegative, early RA. As both currently used diagnostic antibodies (e.g. RF and ACPA antibodies) are involved in the pathogenesis of RA [4], it is interesting to speculate which functionalities the newly identified antigenic targets may have, as this might open up new fields of research into the pathogenesis of RA.

Some of the antigens found have previously been linked to RA. Of the three antigens identified in all groups, increased levels of insulin-like growth factor binding protein 2 (IGFBP2) were found in sera from patients with RA, and these correlate with CRP levels and were speculated to contribute to catabolic states in RA [23]. Heterogeneous nuclear ribonucleoprotein A1 (HNRNPA1) has previously been associated with RA, systemic lupus erythematosus and mixed connective tissue diseases and other rheumatic diseases [15]. A clear connection between N-acetylglucosamine-1-phosphate transferase (GNPTG) and RA is so far unrecognized.

Of the antigens identified specifically in seronegative cohorts, a pathophysiological connection to actin beta (ACTB) is conclusive: autoreactive citrullinated epitopes of beta actin have been identified in synovial fluid in RA [24]. Moreover, pharmacological inhibition of actin cytoskeleton dynamics influences the pathogenicity of fibroblasts in arthritis [25]. A testable hypothesis would thus be that autoantibodies to beta actin might alter the cytoskeleton assembly and functionality of synovial fibroblasts towards an RA phenotype. Further, plasma kallikrein (antibodies to the beta subunit (KLKB1) were identified) is part of a cascade that results in the activation of bradykinins, which are considered to be potent inflammatory mediators with relevance in arthritis [26].

Of the intersecting antigens identified in the mixed cohorts A and B, gelsolin (GSN) was recently identified as a promising urinary biomarker for RA [27]. Increased levels of apolipoprotein A4 (APOA4) have been identified in sera from patients with RA using a proteomic approach [28]. Decreased levels of proline-rich acidic proteins have been identified in patients with RA reporting oral sicca symptoms [29]. The roles of the respective antibodies, like PRAP1-antibodies, have not been investigated in this regard so far. Moreover, dynactin 1 (DCTN1) has been shown to be an integral part of osteoclast formation and function [30]. Finally, even though antibodies to SSB are a hallmark of Sjögren’s syndrome, these antibodies are also found in RA [31].

The identified antibodies to these known targets deserve further functional studies to determine their potential role in the pathogenesis of RA. Of note, most of the antigenic targets identified have not been thoroughly investigated in the context of RA and may thus open up new fields of research. Future studies will adopt the challenge of large-scale screening for post-translationally modified antigens, and to assess the individual performance of the retrieved candidates.

## Conclusions

Comprehensive sequential autoantibody profiling revealed novel, highly interesting, IgG-autoantibodies for future diagnostic assay development and pathophysiological research, especially in seronegative RA.

## Abbreviations

ACPA: anti-citrullinated peptide antibodies
ACR: American College of Rheumatology
ACTB: actin beta
APOA4: apolipoprotein A4
BBA: bead-based assay
BSA: bovine serum albumin
DAS28: Disease Activity Score for 28 joints
ELISA: enzyme-linked immunosorbent assay
EULAR: European League Against Rheumatism
GNPTG: N-acetylglucosamine-1-phosphate transferase
GSN: gelsolin
HNRNP: heterogeneous nuclear ribonucleoprotein
IGFBP: insulin-like growth factor binding protein
KLK: plasma kallikrein
MFI: mean fluorescence activity
PBS: phosphate-buffered saline
PRAP: proline-rich acidic proteins
RA: rheumatoid arthritis
RF: rheumatoid factor
RT: room temperature
TNF: tumor necrosis factor
